# Evaluation of ILAR and PRINTO classifications for juvenile idiopathic arthritis: oligoarticular JIA vs early-onset ANA-positive JIA

**DOI:** 10.1007/s10067-025-07340-z

**Published:** 2025-01-30

**Authors:** Batuhan Küçükali, Çisem Yıldız, Buğra Taygun Gülle, Deniz Gezgin Yıldırım, Sevcan A. Bakkaloğlu

**Affiliations:** 1 Department of Pediatric Rheumatology, Gazi University Faculty of Medicine, 06500 Besevler, Ankara, Turkey; 2 Department of Public Health, Division of Epidemiology, Dokuz Eylül University Faculty of Medicine, Izmir, Turkey

**Keywords:** Classification, ILAR, Juvenile idiopathic arthritis, PRINTO, Treatment response, Uveitis

## Abstract

**Objectives:**

The International League of Associations for Rheumatology (ILAR) juvenile idiopathic arthritis (JIA) classification was revisited by the Pediatric Rheumatology International Trials Organization (PRINTO) in 2018. Classifications should establish uniform groups to assist physicians in providing optimal care. Therefore, we evaluated changes proposed by PRINTO to highlight their impact on forming consistent groups regarding uveitis and treatment responses, particularly focusing on early-onset anti-nuclear antibody (ANA)-positive JIA.

**Methods:**

Pediatric patients diagnosed with JIA according to ILAR and PRINTO classification, with a minimum of 1-year of follow-up, were enrolled, excluding those meeting the exclusion criteria for both the oligoarticular JIA and the early-onset ANA-positive JIA groups.

**Results:**

Among the 139 enrolled patients, 110 (79.1%) had oligoarticular JIA, while 15 (10.8%) had early-onset ANA-positive JIA. The below-age-5 criterion demonstrated the strongest association with uveitis, while the below-age-7 provided similar associations without substantial exclusions (odds ratio (OR) 8.62 [2.50–29.81] vs 7.45 [2.37–26.66]). Patients with a single ANA positivity at a titer ≥ 1/160 and age of onset below 7 had a notably higher risk of new-onset uveitis and biologic DMARD requirement (OR 7.95 [2.37–26.66] and 3.6 [1.42–9.09], respectively).

**Conclusion:**

The inclusion of age of disease onset and ANA positivity with a titer ≥ 1/160 has enhanced uniformity in uveitis risk and treatment response, including failure of conventional synthetic DMARDs. Additionally, a single ANA positivity at a ≥ 1/160 titer rather than requiring two instances yields similar consistency. However, the joint count criteria failed to form consistent groups. PRINTO’s classification places a significant proportion of patients into the “other JIA” group, necessitating further classification for improved clinical utility.

**Table:** 

**Key Points** •*Inclusion of age and ANA positivity criteria increased uniformity among the subgroups*. •*Single ANA positivity at a ≥ 1/160 titer can be sufficient instead of twice*. •*Early utilization of bDMARDs may be beneficial for early-onset ANA-positive JIA group*. •*PRINTO classification must further classify the “other JIA” before being implemented in clinical practice*.

**Supplementary Information:**

The online version contains supplementary material available at 10.1007/s10067-025-07340-z.

## Introduction

Juvenile idiopathic arthritis (JIA) is a diagnosis of exclusion, characterized by chronic arthritis of unknown origin lasting for a minimum duration of 6 weeks [1]. JIA was categorized into seven distinct groups based on the number of joints involved, the presence of extra-articular manifestations, and laboratory markers, including rheumatoid factor (RF) and human leucocyte antigen (HLA) B27, by the International League of Associations for Rheumatology (ILAR) in 2004 [1, 2]. Prior to the ILAR classification, there were two different sets of criteria and definitions for JIA—juvenile chronic arthritis in Europe and juvenile rheumatoid arthritis in the USA—each with varying inclusion and exclusion criteria. Consequently, the ILAR classification has been accepted as an established, uniform classification and terminology for pediatric rheumatologists worldwide. The American College of Rheumatology continues to provide guidelines for JIA according to the ILAR classification [3, 4]. While the ILAR criteria have proven effective in both research and clinical settings, certain categories within the criteria, such as oligoarticular JIA and psoriatic arthritis, have been found to contain heterogeneous groups [5–7]. Additionally, classification based on the number of affected joints has faced criticism [6, 8–10]. Furthermore, a growing body of new data has revealed more information regarding JIA pathophysiology, treatments, and outcomes, highlighting the need to revisit the classification [4, 6, 8, 10–12].

In 2018, the Pediatric Rheumatology International Trials Organization (PRINTO) proposed a revision of the current ILAR classification of JIA, intended for both research and everyday clinical settings [13]. Notably, the revision involved the removal of oligoarticular JIA and the introduction of early-onset anti-nuclear antibody (ANA) positive JIA [13]. While fewer than five involved joints are the only inclusion criterion for oligoarticular JIA, patients must be 6 years old or younger and exhibit ANA positivity at a titer of ≥ 1/160 on two occasions, at least 3 months apart, to meet the criteria for early-onset ANA-positive JIA (Supplementary Information 1). These changes were implemented to establish more consistent groups, particularly concerning treatment responses and the risk of new-onset uveitis [13]. However, no single laboratory test or clinical finding is capable of achieving uniformity among JIA subgroups. Furthermore, this new set of inclusion criteria has not been comprehensively evaluated in large cohort studies for its ability to form consistent groups. Therefore, we conducted an assessment and comparison of the uniformity within both the oligoarticular JIA and early-onset ANA-positive JIA groups in our patient cohort. This analysis focused on treatment responses, uveitis risk, and disease severity scores, aiming to critically evaluate and emphasize the importance and necessity of the changes proposed to the ILAR criteria by PRINTO.

## Methods

**Fig. 1:**
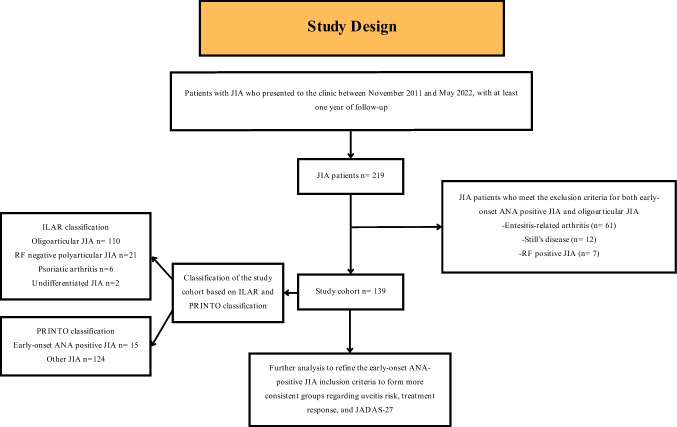
Study design. JIA, juvenile idiopathic arthritis; ANA, anti-nuclear antibody; RF, rheumatoid factor IgM; ILAR, International League of Associations for Rheumatology; PRINTO, Paediatric Rheumatology International Trials Organisation; JADAS-27, Juvenile Arthritis Disease Activity Score-27

Patients, who presented to our Pediatric Rheumatology clinic between November 2011 and May 2022, with a diagnosis of JIA as defined by ILAR and/or PRINTO classification and a minimum 1-year follow-up from disease onset, were enrolled in this retrospective observational study. Patients meeting any exclusion criteria shared by both early-onset ANA-positive JIA and oligoarticular JIA, including systemic JIA, the presence of IgM RF on at least two occasions at least 3 months apart, or enthesitis-related arthritis, were excluded (Fig. 1). Each patient underwent evaluation and treatment in accordance with the most recent American College of Rheumatology guidelines available at the time of their presentation [4, 14, 15]. The diagnosis and management of uveitis were carried out by a specialized ophthalmology team following the standardization of uveitis nomenclature (SUN) working group guidelines [16].

Sex, age at disease onset, number of joints involved, ANA test results, treatment type and duration, treatment response, presence of uveitis, and juvenile arthritis disease activity score-27 (JADAS-27) at disease onset, 6 months, and 1 year after diagnosis were assessed for each patient using electronic medical records and charts. All ANA results were obtained from our laboratory using the immunofluorescent method to eliminate potential laboratory-based differences. Treatments were categorized into conventional synthetic and biological disease-modifying anti-rheumatic drugs (csDMARDs and bDMARDs, respectively).

JADAS-27 was computed by assessing the following variables: physician global rating of overall disease activity; parent/child ratings of well-being and pain; the number of active joints, assessed in 27 joints; and erythrocyte sedimentation rate (ESR), normalized to a 0–10 scale [17]. The JADAS-27 was then determined by summing the scores of its four components, resulting in a global score ranging from 0 to 57 [17]. The cutoff for inactive disease was set at ≤ 1 for all JIA categories, while for minimally active disease, it was set at > 1– ≤ 2 for oligoarticular onset and > 1– ≤ 3.8 for polyarticular onset [18].

All statistical tests were conducted using IBM SPSS software version 29. The Shapiro–Wilk test was used to assess the normality of distributions. For descriptive statistics, the median, 25th, and 75th percentiles were provided for data that did not follow a normal distribution, while number and percentage values were presented for categorical variables. For variables not exhibiting a normal distribution, the Mann–Whitney *U* test was employed; for variable with a normal distribution, the independent sample *t*-test was utilized. Differences in baseline characteristics between groups were evaluated using the Chi-square tests for categorical variables, and odds ratios were calculated. Kendall’s tau test was utilized to evaluate the association due to its appropriateness for ordinal and non-normally distributed data. Correlation coefficients were interpreted as follows: < 0.2 as very weak, 0.2–0.4 as weak, 0.4–0.6 as moderate, 0.6–0.8 as strong, and ≥ 0.8 as very strong. Confidence intervals were calculated at the 95% confidence level, and differences with *p* < 0.05 were considered statistically significant. Missing JADAS-27 data were missing at random and were excluded from the analyses. Notably, the analysis results of the various age inclusion criteria were filtered to focus on the most significant data.

Ethics committee approval for the study was obtained from the Ethics Committee of Gazi University, in accordance with the Declaration of Helsinki, under the identification number E-77082166–604.01–887105. Permission was granted to collect anonymized data without individualized consent, as the study exclusively utilized previously collected data.

## Results

A total of 139 pediatric patients were enrolled in the study after excluding 80 patients from the initial cohort of 219 JIA patients. Among them, 50 (36%) were female. The median follow-up duration was 62 months (interquartile range (IQR): 31–81 months). Among the participants, 110 (79.1%) were diagnosed with oligoarticular JIA according to ILAR classification, while 15 patients (10.8%) were diagnosed with early-onset ANA-positive JIA according to the PRINTO classification. Of the early-onset ANA-positive JIA group, 13 patients were classified as oligoarticular JIA, and two patients were categorized as undifferentiated JIA due to the presence of psoriasis in first-degree relatives based on ILAR criteria.

Clinical characteristics, ANA results, and treatments of the patients are illustrated in Table 1. In the initial ANA tests, ANA positivity was identified in 2 (1.4%) at a titer of 1/640, 9 (6.5%) at 1/320, 23 (16.5%) at 1/160, and 34 (24.5%) at 1/80. A summary of ANA test results is presented in Table 2. Unfortunately, we were unable to provide disease onset JADAS-27 for 13 (9.3%) patients, JADAS-27 at the sixth month after diagnosis for 20 patients (14.4%), and JADAS-27 at 1 year after diagnosis for 31 patients (22.3%) due to missing data in the charts. Notably, two of the patients with missing JADAS-27 at 1 year after treatment and one with missing disease-onset JADAS-27 belonged to the early-onset ANA-positive JIA patient group.

**Table 1:** Clinical features of the patients

Clinical characteristics	Total *n* = 139
Female, *n* (%)	50 (36)
Age at disease onset, median [IQR] (year)	7.0 [3.2;10.3]
Duration of follow-up, median [IQR] (month)	62.0 [31.0;81.0]
ANA titer ≤ 1/80, *n* (%) *	33 (48.5)
ANA titer ≥ 1/160, *n* (%) *	35 (51.5)
ANA titer ≥ 1/160, at least 3 months apart *	25 (36.8)
*ILAR criteria*	
ANA ( +) oligoarticular JIA, *n* (%)	60 (43.2)
ANA ( −) oligoarticular JIA, *n* (%)	50 (36.0)
RF ( −) polyarticular JIA, *n* (%)	21 (15.1)
Psoriatic arthritis, *n* (%)	6 (4.32)
Undifferentiated JIA, *n* (%)	2 (1.44)
*PRINTO criteria*	
Early-onset ANA-positive JIA, *n* (%)	15 (10.8)
Other JIA, *n* (%)	124 (89.2)
*Number of involved joints at disease onset*	
One joint, *n* (%)	25 (18.0)
Two to four joints, *n* (%)	89 (64.0)
More than four joints, *n* (%)	25 (18.0)
*csDMARDs*	
Methotrexate, *n* (%)	118 (87.4)
Sulfasalazine, *n* (%)	7 (5.1)
*bDMARDs*	
Etanercept, *n* (%)	40 (29.9)
Adalimumab, *n* (%)	18 (12.9)
Tocilizumab, *n* (%)	3 (2.6)
Infliximab, *n* (%)	1 (0.7)
Uveitis, *n* (%)	14 (10.1)

*IQR* interquartile range, *JIA* juvenile idiopathic arthritis, *ILAR* International League of Associations for Rheumatology, *ANA* anti-nuclear antibody, *PRINTO* Paediatric Rheumatology International Trials Organisation, *csDMARDs* conventional disease modifying anti-rheumatic drugs, *bDMARDs* biologic disease modifying anti-rheumatic drugs

^*^Only the percentages of ANA-positive individuals are provided. The prevalence of ANA positivity is 48.9% (*n* = 68)

**Table 2:** ANA test results of the patients

Initial ANA result (*n* = 68)	Second ANA result (*n* = 45)					
	Negative	1/80 titer positive	1/160 titer positive	1/320 titer positive	1/640 titer positive	Not performed
1/80 titer positive (*n* = 34)	2 (5.9%)	13 (38.2%)	1 (3%)	-	-	18 (52.9%)
1/160 titer positive (*n* = 23)	1 (4.4%)	3 (13%)	13 (56.5%)	1 (4.4%)	-	5 (21.7%)
1/320 titer positive (*n* = 9)	-	-	7 (77.8%)	2 (22.2%)	-	-
1/640 titer positive (*n* = 2)	-	-	1 (50%)	-	1 (50%)	-

*ANA* anti-nuclear antibody

JADAS-27 for the patients and comparisons based on age and ANA status regarding JADAS-27 are summarized in Table 3. At 1 year after diagnosis, lower median JADAS-27 were observed only in patients aged below 7 years with ANA positivity at a titer of ≥ 1/80 compared to the excluded patients. Meanwhile, JADAS-27 assessments at 1 year after diagnosis in all other groups were similar to each other. None of the groups showed uniform results in terms of minimally active disease rates at 1 year after diagnosis compared to others.

**Table 3:** JADAS-27 across different classifications

Classification of patients	JADAS-27 at disease onset *n* = 126 median [IQR]	JADAS-27 at sixth month *n* = 119 median [IQR]	JADAS-27 at 1 year *n* = 108 median [IQR]	Inactive or minimally active disease at 1 year according to JADAS27 *n* = 108 (%)
Oligoarticular JIA *n* = 110	11 *	2.5	1	62
	**(9; 13)**	[0; 7]	(0; 4)	(69.7)
Others *n* = 29	16 *	4	1	13
	**(12; 21)**	(1; 12)	(0; 7)	68.4)
Early-onset ANA-positive JIA *n* = 15	12	5.5 *	1	9
	(11.5; 16)	(**2.5; 10.25**)	(0; 4)	(69.2)
Others *n* = 124	12	2 *	1	66
	(9; 14.7)	(**0; 7**)	(0; 3.5)	(69.5)
ANA positivity 1/160 titer ≥ and age < 7 *n* = 23	12	5	0.5	16
	(10; 16)	(0.5; 8.50)	(0; 2)	(80)
Others *n* = 116	12	2.5	1	59
	(9; 14.5)	[0; 7]	[0; 5]	(67)
ANA positivity 1/160 titer ≥ and age < 5 *n* = 16	12	5	0	11
	(10; 16)	[0; 10]	[0; 3]	(73.3)
Others *n* = 123	12	3	1	64
	[9; 15]	[0; 7]	[0; 4,5]	(68.8)
ANA positivity 1/160 titer ≥ and age < 9 *n* = 26	12	5	0.5	17
	[9.2; 15.5]	[0; 7.7]	[0; 2.2]	(77.3)
Others *n* = 113	12	3	1	58
	[9; 15]	[0; 7]	[0; 5]	(67.4)
ANA positivity 1/80 titer ≥ and age < 7 *n* = 40	12	3	0 *	32
	[9; 16]	[0; 7]	**[0; 2]**	(80)
Others *n* = 99	12	3.5	1 *	43
	[9; 16]	[0; 7]	**[0; 6]**	(63.2)
Monoarticular onset *n* = 25	9*	0*	0	17
	**[7; 12]**	**[0; 1]**	[0; 1]	(85)
Others *n* = 114	12*	5*	1	58
	**[9; 16]**	**[1; 8]**	[0; 4.7]	(65.4)
All patients	12	3	1	75
	[9; 15]	[0; 7]	[0; 4]	(69.4)

*JIA* juvenile idiopathic arthritis, *JADAS-27* Juvenile Arthritis Disease Activity Score-27, *ANA* anti-nuclear antibody

^*^p value < 0.05

Sulfasalazine treatment was administered to 5.1% patients, categorized as follows: five with ANA negative oligoarticular JIA, one with ANA-positive oligoarticular JIA (1/80 titer), and one with RF negative polyarticular JIA. Among the patients, 40 were treated with one bDMARD, while 3 required two different bDMARDs and another 3 needed three different bDMARDs (including two different tumor necrosis factor-alpha (TNF-α) inhibitors and tocilizumab) to achieve inactive disease. Additionally, 3 patients in the early-onset ANA-positive JIA group required a bDMARD switch due to new onset uveitis. Uveitis emerged in 14 participants (10.1%) within the study period.

When participants were categorized into two groups—those with ANA positivity at a titer of 1/160 or higher and aged younger than 7, versus others—it was evident that the risk of new onset uveitis and the requirement of bDMARDs were higher in the ANA-positive group (with respective OR [95% CI] of 7.95 [2.37–26.66] and 3.60 [1.42–9.09]). However, no linear association was observed between ANA titer and uveitis (correlation coefficient, 0.182; *p* value, 0.115). Table 4 illustrates the potential risks of uveitis and the requirement of bDMARDs across various conceivable categorizations.

**Table 4:** Uveitis risk and bDMARD requirements in different groups based on ANA test results

Classification of patients	Presence of uveitis (*n* = 14)		bDMARDs requirement (*n* = 49)	
	***n* (%)**	**OR [CI 95%]**	***n* (%)**	**OR [CI 95%]**
Oligoarticular JIA (ILAR) (*n* = 110)	13 (11.8)	3.46 [0.43–27.8]	37 (33.6)	0.71 [0.31–1.66]
Others (*n* = 29)	1 (3.4)		12 (41.4)	
Early-onset ANA + JIA (PRINTO) (*n* = 15)	4 (26.7)	4.60 [1,21–17.42] *	9 (60)	3.15 [1.05–9.46] *
Others (PRINTO) (*n* = 124)	10 (7.3)		40 (32.3)	
ANA positivity ≥ 1/160 titer and age < 7 (*n* = 23)	7 (30.4)	7.95 [2.37–26,66] *	14 (60.9)	3.6 [1.42–9.09] *
Others (*n* = 116)	7 (5.2)		35 (30.2)	
ANA positivity ≤ 1/80 titer (two times) and age < 7 (*n* = 27)	5 (19.2)	3.10 [0.92–10.40]	13 (48.1)	1.96 [0.84–4.60]
Others (*n* = 112)	9 (7.1)		36 (32.1)	
ANA positivity ≤ 1/80 titer and age < 7 (*n* = 45)	9 (20.5)	5.79 [1.67–20.01] *	19 (42.2)	1.56 [0.75–3.25]
Others (*n* = 94)	4 (4.3)		30 (31.9)	
ANA positivity ≥ 1/160 titer (*n* = 35)	9 (22.9)	2.86 [0.69–11.93]	15 (42.9)	2 [0.72–5.53]
ANA positivity 1/80 titer (*n* = 33)	3 (9.4)		9 (27.3)	
ANA positivity 1/320 or 1/640 titer (*n* = 12)	3 (25)	1.96 [0.43–8.83]	7 (58.3)	3.21 [0.89–11.57]
ANA positivity 1/80 or 1/160 titer (*n* = 56)	8 (14.5)		17 (30.4)	
> 1 joint (*n* = 114)	12 (9.7)	1.24 [0.26–5.98]	44 (38.6)	2.51 [0.88–7.19]
1 joint (*n* = 25)	2 (8)		5 (20)	

*JIA* juvenile idiopathic arthritis, *ILAR* International League of Associations for Rheumatology, *OR* odds ratio, *CI* confidential interval, *ANA* anti-nuclear antibody, *PRINTO* Paediatric Rheumatology International Trials Organisation

^*^p value < 0.05

When participants with ANA positivity at a titer of 1/160 or higher were grouped with different age cutoffs, an age < 5 years was associated with the highest risk of uveitis and bDMARD requirement (OR for uveitis 8.62 [2.50–29.81], OR for bDMARD utilization 4.92 [1.60–15.15]). No linear association was found between age at disease onset and bDMARD requirement (Kendall’s Tau, *p* = 0.065; correlation coefficient, − 0.129). The relationship between different age groups and the risk of uveitis and the utilization of bDMARDs is presented in Table 5.

**Table 5:** Uveitis risk and bDMARD requirements in different groups based on age

Classification of patients	Presence of uveitis (*n* = 14)		bDMARDs requirement (*n* = 49)	
	***n* (%)**	**OR [CI 95%]**	***n* (%)**	**OR [CI 95%]**
ANA positivity ≥ 1/160 titer and age < 9 (*n* = 26)	7 (26.9)	5.58 [1.76–17.72] *	14 (53.8)	2.6 [1.09–6.19] *
Others (*n* = 113)	7 (6.2)		35 (31)	
ANA positivity ≥ 1/160 titer and age < 8 (*n* = 24)	7 (29.2)	6.35 [1.98–20.83] *	14 (58.3)	3.2 [1.30–7.90] *
Others (*n* = 115)	7 (6.1)		35 (30.4)	
ANA positivity ≥ 1/160 titer and age < 7 (*n* = 23)	7 (30.4)	7.45 [2.37–26,66] *	14 (60.9)	3.60 [1.42–9.09] *
Others (*n* = 116)	7 (6)		35 (30.2)	
ANA positivity ≥ 1/160 titer and age < 6 (*n* = 21)	7 (33.3)	7.93 [2.42–25.96] *	13 (61.9)	3.70 [1.41–9.71] *
Others (*n* = 118)	7 (5.9)		36 (30.5)	
ANA titer ≥ 1/160 titer and age < 5 (*n* = 16)	6 (37.5)	8.62 [2.50–29.81] *	11 (68.8)	4.92 [1.60–15.15] *
Others (*n* = 123)	8 (6.5)		38 (30.9)	
ANA titer ≥ 1/160 titer and age < 4 (*n* = 13)	5 (38.5)	8.12 [2.20–30.02] *	9 (69.2)	4.84 [1.41–16.65] *
Others (*n* = 126)	9 (7.1)		40 (31.7)	

*JIA* juvenile idiopathic arthritis, *OR* odds ratio, *CI* confidential interval, *ANA* anti-nuclear antibody

^*^p value < 0.05

## Discussion

The PRINTO classification’s early-onset ANA-positive JIA subgroup demonstrated improved uniformity regarding uveitis risk, JADAS27, and treatment responses compared to the oligoarticular JIA subgroup defined by ILAR. However, further evaluation of each inclusion criterion for early-onset ANA-positive JIA subgroup indicated that single ANA positivity may achieve similar homogeneity, while lowering the age limit could result in unnecessary patient exclusions without offering substantial gains in uniformity.

ANA positivity has been linked to various autoimmune diseases, particularly at high titers, whereas 1/40–1/80 are frequently identified in the healthy population [19]. ANA positivity in JIA patients has been associated with uveitis [20]. However, the clinical implications of ANA titers have not been extensively studied in JIA patients [21]. Furthermore, the threshold for considering ANA positivity has varied among studies, with most studies adopting a titer of ≥ 1/80 as the cutoff [22–24]. According to our findings, in patients younger than 7 years old, ANA positivity at a ≥ 1/80 titer is associated with uveitis, but ANA positivity at a ≥ 1/160 titer shows the strongest link to uveitis (OR 5.79 [1.67–20.01] vs 7.95 [2.37–26.66]). However, no clear linear correlation was observed between higher ANA titers and the risk of uveitis among all patients (ANA titers > 1/160 vs ≤ 1/160 titers, OR 1.96 [0.43–8.83]). This lack of correlation may be attributed to the limited number of patients with ANA positivity at > 1/160 titers (*n* = 12). Additionally, ANA positivity at > 1/160 titers should raise suspicion of autoimmune diseases with joint involvement, such as connective tissue diseases. Establishing a cutoff for ANA positivity is essential to reduce heterogeneity between studies. Accepting a cut-off titer of 1/80 may lead to overrepresentation in the high uveitis risk group, whereas considering > 1/160 titers as the cutoff may exclude patients at high risk. Therefore, based on our findings, ANA positivity at a titer of ≥ 1/160 performs better as an inclusion criterion.

Transient ANA positivity may be encountered under various conditions, particularly during viral infections [25]. However, studies have shown that ANA positivity, especially at ≥ 1/160 titers, can persist without substantial changes in titers, even in healthy children [26]. This aligns with our findings, where only 31.1% of patients demonstrated a decrease in ANA positivity titers at the second test. Moreover, 87.3% patients whose initial ANA positivity was at ≥ 1/160 titer remained ANA positive at the same or higher titer during second test. Furthermore, the association between uveitis and ANA positivity at a ≥ 1/160 titer on two separate occasions at least three months apart was not stronger than the association observed with a single ANA positivity at a ≥ 1/160 titer in children aged younger than 7 (OR 4.60 [1.21–17.42] vs 7.95 [2.37–26.66]). In the light of our findings and the existing literature, we recommend that a single ANA positivity at a titer of ≥ 1/160 is sufficient to assess uveitis risk in JIA patients [27]. Notably, JIA treatments, particularly TNF-α inhibitors, may influence ANA test results [28]. Therefore, imposing a requirement for a second ANA test may increase healthcare burdens, delay patient classification, necessitate additional blood sampling, and potentially postpone informing patients about their condition and prognosis.

The ILAR classification’s number of involved joints criterion has faced criticism due to its poor predictive value for prognosis [5, 6, 8]. Moreover, elucidation of JIA pathophysiology has resulted in the addition of age and ANA positivity criteria, as well as exclusion of the involved joint count criterion in the PRINTO classification [11, 13, 29, 30]. Since joint involvement criterion rely on the patient’s symptoms and physical examination, which may be misleading, and the recent integration of musculoskeletal ultrasonography into clinical practice has allowed for the detection of subclinical articular inflammation, omitting the initial active joint count from the classification criteria may be a rational decision [31, 32]. Furthermore, the oligoarticular JIA group failed to predict uveitis in our study (OR 3.46 [0.43–27.8]). Zulian et al. proposed that persistent monoarticular JIA constitutes a distinct group with a lower uveitis risk, ANA positivity, and better long-term outcomes [9]. However, in our study, monoarticular onset was not associated with a change in uveitis risk (monoarticular vs others—uveitis rates 9.7% vs 8%, OR 1.24 [0.26–5.98]). While JADAS-27 at disease onset and sixth months were lower, as expected due to fewer swollen joints, scores at 1 year after diagnosis or rates of inactive/minimally active disease did not significantly differ from others (median (IQR) JADAS-27 at 1 year of monoarticular onset vs. others, 0 (0;1) vs 1 (0;4.7); *p* value, 0.066). However, the highest inactive/minimally active disease rate at 1 year after diagnosis was identified in the monoarticular onset group (85%; *p*, 0.094). Furthermore, the requirement for bDMARDs was higher (38.6% vs 20%). However, Zulian et al. noted that 39.2% of monoarticular onset patients switch to the oligoarticular form, and our data is based only on the disease onset involved joint count [9]. Therefore, it may not be accurate to compare our data directly with theirs.

Juvenile idiopathic arthritis presents with well-known clinical phenotypes, and the age of disease onset plays a crucial role in defining these phenotypes [6, 33]. Additionally, the significance of this association is supported by identical B-cell signatures observed in JIA patients based on age, irrespective of the number of affected joints, as demonstrated by Barnes et al. [11]. In our study, we identified that the strongest association with uveitis occurred with an age cutoff of < 5 for patients with a single ANA positivity at a ≥ 1/160 titer. However, there was no substantial difference in risk between age cutoffs of < 5 and < 7 (OR 8.62 [2.50–29.81] vs 7.95 [2.37–26.66]). Additionally, increasing the age cutoff to below 9 years only increased the group count by 13% (*n* = 3), while significantly reducing uveitis risk (OR 5.58 [1.76–17.72] vs 8.62 [2.50–29.81]). Based on our findings, an age cutoff below 7 for patients with ANA positivity at a ≥ 1/160 titer appears to be a feasible choice, providing the strongest risk for uveitis without excluding a substantial portion of patients.

Treatment response is an important aspect of clinical classifications. Therefore, we evaluated the treatment requirements of our patients to determine whether any classification group could achieve similar treatment response consistency, thereby aiding treatment suggestions. Our findings indicate that the risk of csDMARD failure is highest in patients with a single ANA positivity at a ≥ 1/160 titer in children aged younger than 7 years. Notably, a single or twice ANA positivity at a ≥ 1/80 titer was not found to be associated with the requirement bDMARDs. Moreover, the age of onset was associated with bDMARD requirement rates, with the highest rates observed in patients below 5 years of age (ANA positivity ≥ 1/160 titer and age < 7, *n* = 23 vs. ANA positivity ≥ 1/160 titer and age < 5, *n* = 13; OR 3.60 [1.42–9.09] vs. 4.92 [1.60–15.15]). Therefore, utilizing early bDMARD treatment strategies may be reasonable for children below 5 years of age with a single ANA positivity at a ≥ 1/160 titer.

JADAS-27 and disease activity at 1 year after diagnosis play an important role in classification and help physicians predict treatment responses [34]. The oligoarticular JIA group exhibited lower JADAS-27 at disease onset, as expected, due to a lower count of involved joints. However, JADAS-27 and rates of inactive/minimally active disease at 1 year after diagnosis were similar to those observed in other groups. In contrast, the early-onset ANA-positive JIA group showed worse JADAS-27 at the sixth month but similar assessments 1 year after diagnosis. This observation could be attributed to the high risk of csDMARD failure and the requirement for bDMARD treatment in the early-onset ANA-positive group. Early initiation of bDMARDs or bypassing csDMARDs could be a feasible treatment plan for these patients if they exhibit further unfavorable prognostic findings, such as ankle involvement or high acute phase reactants [34, 35].

According to PRINTO, 124 patients were classified into the “other JIA” group, whereas ILAR categorized only 2 patients into the undifferentiated JIA group, which is compatible with the findings of the study by Lee et al. [36]. While it is crucial to group patients with similar prognoses together, leaving a majority of the patients in undifferentiated groups may diminish the validity and applicability of the classification in clinical practice. Therefore, despite PRINTO taking a significant step forward in creating standardized classification criteria, it places a substantial portion of patients in the “other JIA” group, leaving them in obscurity. However, PRINTO has stated that further classification attempts for the “other JIA” group will be made based on specific genetic tests aligned with pathophysiology [13].

## Limitations

Early-onset ANA-positive JIA group comprised 15 patients, while 124 patients were categorized in the “other JIA” group. The limited number of early-onset ANA-positive JIA patients may be attributed to the absence of second ANA tests in 18 patients with a titer of 1/80 ANA positivity and 5 patients with a titer of 1/160 ANA positivity. This was because the ILAR criteria did not mandate second ANA tests, and those who underwent multiple tests often had high-titer (> 1/160 titer) positivity in initial test or symptoms suggestive of connective tissue diseases. Despite this, among patients with a 1/80 ANA titer who underwent a second test, only 1 (6.2%) exhibited 1/160 ANA positivity in follow-up testing. This indicates that 1/80 ANA positivity rarely progresses to higher titers. Therefore, although many patients lacked a second ANA test, most of these patients would not have met the criteria for inclusion in the early-onset ANA-positive JIA group, and their absence is unlikely to have significantly impacted the study.

Juvenile idiopathic arthritis associated uveitis is a major cause of JIA-associated disability [37, 38]. However, the severity of uveitis and its complications, such as band keratopathy, maculopathy, and optic neuropathy, also significantly affects the quality of life of JIA patients. In our study, we focused solely on the presence of uveitis without addressing its severity or complications. Therefore, further studies should investigate these critical aspects of uveitis.

JADAS-27 provides a substantial overview of current disease activity in JIA patients [17]. However, JADAS does not account for articular damage, joint function loss, or changes in quality of life. In our study, JADAS-27 was used for joint assessments. Nonetheless, future research should incorporate radiological imaging to demonstrate bone and cartilage damage and employ functional questionnaires to offer a more comprehensive, multidimensional evaluation of joint prognosis.

## Conclusion

Juvenile idiopathic arthritis classifications should aim to establish homogeneous groups in terms of treatment responses, complications, particularly uveitis, and disease prognosis. Including the age of disease onset and ANA positivity at a titer ≥ 1/160 has improved uniformity in assessing uveitis risk and treatment response. Although the PRINTO classification criteria mandate ANA positivity at a titer ≥ 1/160 on two separate occasions at least 3 months apart, a single ANA positivity at a ≥ 1/160 titer was found to yield similar results. Uveitis risk was highest in children under 5 years of age. However, a < 7 years age of disease onset cutoff is feasible, providing significant consistency across groups regarding uveitis risk without excluding a substantial proportion of patients. In terms of treatment responses, the early-onset ANA-positive JIA group was associated with a higher risk of csDMARDs failure. Therefore, early initiation of bDMARDs may be beneficial for this group. Additionally, the number of involved joint count criterion failed to form uniform groups and does not align with the disease’s pathophysiology. The newly proposed PRINTO classification, particularly its early-onset ANA-positive JIA subgroup, represents a significant advancement in the light of recently elucidated disease-causing molecular pathways. However, it places a substantial proportion of patients to the “other JIA” group, making utilization of the PRINTO classification challenging in daily practice. Further classification of the “other JIA” subgroup and prospective validation studies are necessary to enhance its effectiveness.

## Supplementary Information

Below is the link to the electronic supplementary material.

Supplementary file1 (DOCX 98 KB)

## Data Availability

The data underlying this article will be made available upon reasonable request to the corresponding author.
