# Supplementary material for: Evaluation of ILAR and PRINTO classifications for juvenile idiopathic arthritis: oligoarticular JIA vs early-onset ANA-positive JIA
Source: Clin Rheumatol. 2025 Jan 30;44(3):1307–16. doi: 10.1007/s10067-025-07340-z (PMC11865100; doi:10.1007/s10067-025-07340-z)
Supplement: Supplementary file 1 — Supplementary file1 (DOCX 98 KB) [file 10067_2025_7340_MOESM1_ESM.docx]

**Supplementary Information 1.** The oligoarticular JIA and the early-onset ANA positive JIA classification criteria

**
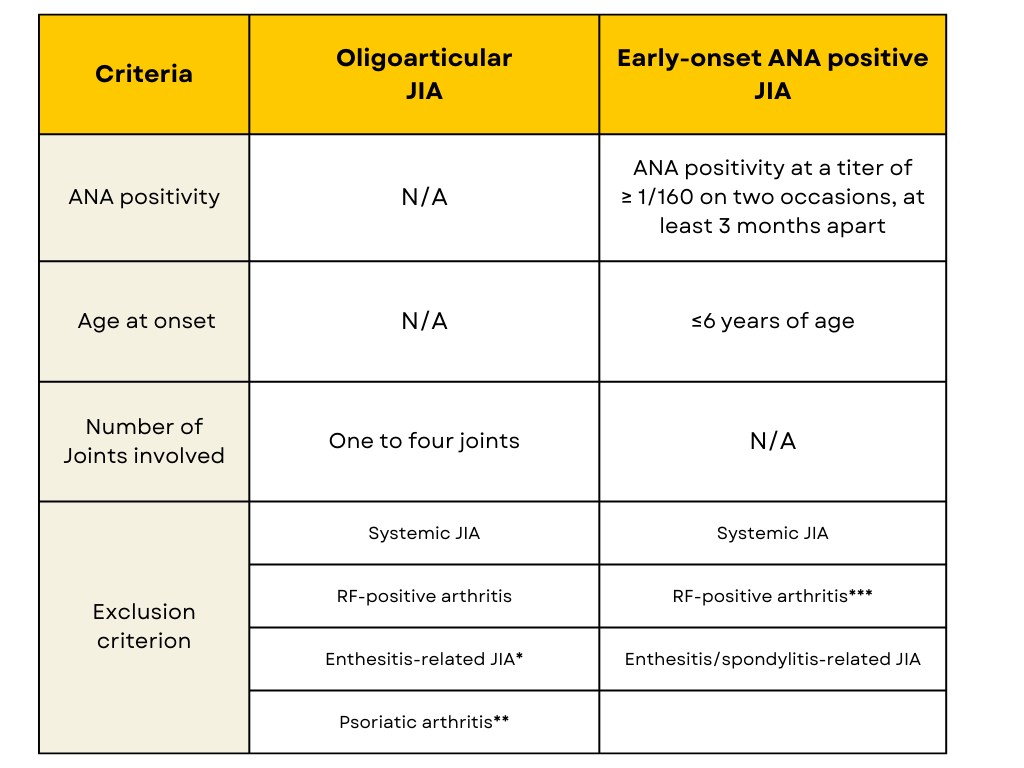
**

* Arthritis in an HLA-B27 positive male beginning after the 6th birthday or Ankylosing spondylitis, enthesitis related arthritis, sacroiliitis with inflammatory bowel disease, Reiter’s syndrome, or acute anterior uveitis, or a history of one of these disorders in a first-degree relative, ** Psoriasis or a history of psoriasis in the patient or first degree relative, *** 2 positive tests for RF at least 3 months apart or at least 1 positive test for antibodies to cyclic citrullinated peptide. JIA: Juvenile idiopathic arthritis, ANA: Anti-nuclear antibody, RF: Rheumatoid Factor IgM
